# Cerebellar Patterning Defects in Mutant Mice

**DOI:** 10.3389/fnins.2021.787425

**Published:** 2021-12-08

**Authors:** Richard Hawkes

**Affiliations:** Department of Cell Biology, Cumming School of Medicine, Anatomy and Hotchkiss Brain Institute, University of Calgary, Calgary, AB, Canada

**Keywords:** cerebellar pattern formation Purkinje cell, granule cell, transverse zone, stripe, pattern formation, cerebellar development

## Abstract

The cerebellar cortex is highly compartmentalized and serves as a remarkable model for pattern formation throughout the brain. In brief, the adult cerebellar cortex is subdivided into five anteroposterior units—transverse zones—and subsequently, each zone is divided into ∼20 parasagittal stripes. Zone-and-stripe pattern formation involves the interplay of two parallel developmental pathways—one for inhibitory neurons, the second for excitatory. In the inhibitory pathway, progenitor cells of the 4th ventricle generate the Purkinje cells and inhibitory interneurons. In the excitatory pathway, progenitor cells in the upper rhombic lip give rise to the external granular layer, and subsequently to the granular layer of the adult. Both the excitatory and inhibitory developmental pathways are spatially patterned and the interactions of the two generate the complex topography of the adult. This review briefly describes the cellular and molecular mechanisms that underly zone-and-stripe development with a particular focus on mutations known to interfere with normal cerebellar development and the light they cast on the mechanisms of pattern formation.

## Introduction

This review addresses the fundamental compartmentation of the cerebellar cortex into transverse zones and parasagittal stripes. On the other hand, the review does not address secondary aspects of patterning such as the lobulation of the cortex (e.g., Legué et al., 2015) and the regional restriction of the afferent terminal fields and various interneurons (e.g., Consalez and Hawkes, 2013). The key perspective is the light cast on patterning by mouse mutant phenotypes.

Briefly, the cerebellar cortex is highly patterned into hundreds, if not thousands, of genetically predetermined quanta (reviewed in Apps and Hawkes, 2009; Armstrong and Hawkes, 2014; Leto et al., 2016; Apps et al., 2018). The earliest pattern to form is an anteroposterior array of transverse zones defined by boundaries between Purkinje cell (PC) domains (Ozol et al., 1999). Five zones are reproducibly identified to date in all birds and mammals: the anterior zone (AZ), central zone (CZ—further subdivided into anterior and posterior domains: Marzban et al., 2008), posterior zone (PZ) and nodular zone (NZ). Similar transverse boundaries are also found in the granular layer.

Each transverse zone is subdivided from medial to lateral into parasagittal stripes. This patterning is most clearly seen in the adult in the PC expression patterns of numerous molecules. For example, the AZ and PZ show alternating striped arrays of PC expression for zebrin II/aldolase C (= zebrin II+, Brochu et al., 1990; Ahn et al., 1994) or the zebrin II- subset (e.g., phospholipase (PL)Cβ4 (Sarna et al., 2006) or the transcription factor Early B-cell Factor 2 (Ebf2, Croci et al., 2006). In contrast, the CZ and NZ are uniformly zebrin II + but stripes are seen in other expression patterns [e.g., heat shock protein (HSP)25±, Armstrong et al., 2001 or HNK-1, Marzban et al., 2004]. In all zones, the zone-and-stripe patterns are highly reproducible between individuals and conserved across species (e.g., Sillitoe et al., 2005), but interspecific size differences are typical, reflecting a species’ particular lifestyle. For example, mosaic evolution results in the CZ being especially large in bats (Kim et al., 2009), where it subserves echolocation and vision, whereas it is especially small in blind species such as the naked mole rat (Marzban et al., 2011) or star-nosed mole (Marzban et al., 2015; see also Legué et al., 2015). In parallel to the patterning of the PCs, the granular layer is similarly patterned into transverse domains that align with the PC transverse zones (e.g., Sillitoe et al., 2003).

In brief, the zone-and-stripe pattern forms during development via two separate but interacting pathways (reviewed in Leto et al., 2016). In brief, the inhibitory neurons—in particular the PCs—are born in the ventricular zone of the fourth ventricle (VZ: E10-E13 in mice). Postmitotic PCs migrate first to the cerebellar plate which subsequently disperses to form a stereotyped array of clusters (E14-E18), which are the topographic organizing centers that pattern the afferent connections and the interneurons (reviewed in Consalez and Hawkes, 2013; Apps et al., 2018). In parallel to the PC patterning, the granule cells are generated from granule cell precursors (G) in the upper rhombic lip (URL), which divide extensively and spread to cover the cerebellar anlage as the external granular layer (EGL) by E18. Thus, by birth the fundamental zone-and-stripe architecture has been laid down. Subsequently the embryonic cerebellum transforms into the adult: between ∼E18-P20 the granule cells in the EGL and the PC clusters all disperse to form the elaborate adult zone-and-stripe array.

Dozens of mutations are known that impede normal cerebellar development and many show abnormal morphology. The underlying disruptions are of two kinds: “patterning” defects—mutations that disturb normal pattern formation; and “patterned” defects—those which are secondary consequences of the underlying abnormal patterns. A pertinent analogy might be the bud and the flower. Mutations acting on bud formation might result, for example, in missing petals; in contrast, in mutations acting on the transformation of the bud to the flower, all the normal components would be present but in some way distorted. Almost all cerebellar mutants are of the second kind—all normal elements are present but disorganized. Indeed, it is arguable that mutations of the first kind have never been unambiguously identified.

## Patterned Phenotypes in the Inhibitory Purkinje Cell Development Pathway

PC pattern formation occurs in four distinct phases during which complexity steadily increases: PC birth in the ventricular zone of the VZ; migration to the cerebellar plate; dispersal of the cerebellar plate into a stereotypes array of PC clusters; and dispersal of the cluster array to reveal the mature zone-and-stripe architecture. In the interests of space, the patterning events that precede the formation of the cerebellar anlage (∼pre-E10) are not considered here (for an overview of PC patterning with numerous useful illustrations, see Leto et al., 2016).

Precisely when the zone-and-stripe pattern is first specified in cerebellar development is uncertain. There are several candidate stages at which aspects of the adult architecture might be specified: in the VZ; in the cerebellar plate; in the transformation of the cerebellar plate into the embryonic cluster array; and during the metamorphosis of the cluster array to the adult stripes. In all likelihood, stages in pattern formation involves several.

### From Ventricular Zone to Cerebellar Plate

All PCs are generated between E10-E13 in a region of the 4th VZ, specified by the expression of the basic helix-loop-helix factor pancreatic transcription factor 1a (*Ptf1a*, Hoshino et al., 2005; reviewed in Dastjerdi et al., 2012). When *Ptf1a* is disrupted by a downstream transgene insertion (the mutant mouse *cerebelless*; *cbll*; Hoshino et al., 2005) the result is a comprehensive ablation of the entire cerebellar GABAergic phenotype. The newborn post-mitotic PCs migrate dorsally from the VZ to form the cerebellar plate by about E13.

It is possible that some cerebellar patterning is already patterned in the VZ (a “protomap”; Rakic, 1988). Whether or not a protomap in present there are certainly mediolateral subdomains in the *Ptf1a* + VZ (e.g., Chizhikov et al., 2006; Zordan et al., 2008) but how, if at all, these relate to patterning, is unknown. The proposition that patterning of the cerebellar cortex starts early in development is supported by studies in mice null for the zinc finger protein Zfp423. *Zfp423* codes for a transcription factor with a link to the ciliopathy Joubert syndrome. *Zfp423* is expressed in the VZ from E11 onward and the consequences of its mutation include disruption of normal PC generation. In homozygous null alleles the cerebellum is deformed and has prominent vermian hypoplasia (Warming et al., 2006). Engineered mouse lines with targeted domain deletions (*notably Zfp423*Δ*9-20;* Casoni et al., 2017) show more subtle PC phenotypes. In particular, *Zfp423*Δ*9* shows a significant loss of PCs focussed on the late-born zebrin II-/Ebf2 + population. The upshot is that in *Zfp423*Δ*9-20* the adult PC stripe pattern is abnormal, with reduced size (but not number) of the zebrin II- stripes. In contrast, in the *Zfp423*Δ*28-30* allele there is also PC loss, but subtypes are not differentially affected.

Generally speaking, one aspect of the adult phenotype is certainly specified during the VZ-cerebellar plate (E10-E13) period—the adult PC zebrin II ± phenotype. In general, the future zebrin II + PCs are born first (∼E10-E11.5) followed by the zebrin II- population (∼E11.5-E13; Hashimoto and Mikoshiba, 2003). These two populations can already be distinguished in the cerebellar plate by expression data. For example, the transcription factor Ebf2 is a regulator of the zebrin II- PC phenotype (Croci et al., 2006; Chung et al., 2008). During development (from E12 to adult) *Ebf2* expression distinguishes two PC phenotypes, with the early born *Ebf2-* PCs becoming zebrin II + in the adult and the later-born *Ebf2* + PCs destined to become zebrin II-. The two PC subpopulations stack in the cerebellar plate such that the Ebf2- PCs are preferentially located dorsally. The pathway to zebrin II ± phenotype specification is poorly understood. One model involves Neurogenin (*Neurog*)1/2 and evokes a *Ptf1a*→*Neurog1/*2→*Ebf2* regulatory network (reminiscent of frontal cortex development: e.g., Schuurmans et al., 2004; Zordan et al., 2008; Lundell et al., 2009). The hypothesis is that *Ptf1*a upregulates *Neurog1/2* in the late-born PC progenitors. In turn, *Neurog1/2* upregulates *Ebf2* and thereby downregulates the zebrin II + phenotype. Conversely, the early born PC cohort (E10-E11.5) expresses neither *Neurog1/2* nor *Ebf2* and therefore becomes zebrin II + in the adult. Consequently, in mice where *Ebf2* is ablated a complex cerebellar null phenotype results, in which some PCs die and many others undergo a transdifferentiation such that adult PC stripes that are normally Ebf2 + /zebrin II- ectopically express markers normally restricted to the zebrin II + stripes, resulting in an abnormal transdifferentiated Ebf2 + /zebrin II + phenotype; Croci et al., 2006; Chung et al., 2008). This suggests that PC subtype specification is achieved through *Ebf2* acting as a repressor of the zebrin II + phenotype. Indeed, the *Ebf2* null mutation is the only known genetic manipulation to subvert PC subtype specification. These data have two important implications for patterning. First, it is clear that at least two distinct PC classes (zebrin II + /Ebf2- and zebrin II-/Ebf2 +) are specified as soon as E13. Secondly, at least some positional information is also specified in in the sense that late-born Ebf2 + PCs reliably end up in the adult zebrin II- stripes.

### From Cerebellar Plate to Embryonic Purkinje Cell Clusters

Between E14-E18 the cerebellar plate migrates dorsally and transforms into ∼54 reproducible PC clusters (Fujita et al., 2012; Vibulyaseck et al., 2017) with multiple expression profiles (Wassef and Sotelo, 1985; reviewed in Dastjerdi et al., 2012). PC clusters are the forerunners of the adult parasagittal stripes and also serve as organizing centers for the topography of other cerebellar components—interneurons, afferents etc. (e.g., Consalez and Hawkes, 2013; Apps et al., 2018). The quantitative relationship between individual clusters and stripes is complex. In some cases, a single cluster transforms into a single stripe but in other cases one cluster splits into multiple stripes (e.g., Armstrong et al., 2000; Armstrong and Hawkes, 2001; Sillitoe et al., 2009; see also Fujita et al., 2012; Vibulyaseck et al., 2017). Furthermore, several clusters may merge into a single stripe [although more likely, where several clusters combine, there are several contiguous stripes with similar marker expression—e.g., the zebrin II- stripes of the anterior vermis (Ozol et al., 1999; Marzban et al., 2007)]. Whatever the ultimate case, differential expression by PC cluster subtypes is seen already at E14 (e.g., the Olfactory Marker Protein-*lacZ* transgene: Nunzi et al., 1999) and by E18 multiple cerebellar PC phenotypes are present (e.g., reviewed in Dastjerdi et al., 2012; Armstrong and Hawkes, 2014). The mechanisms that convert the cerebellar plate into to the perinatal cluster array are not understood. There is some evidence that the transformation is guided by homophilic interactions between cadherins (e.g., Redies et al., 2011) and/or ephrins (which, in turn, are linked to the reelin signaling pathway—see below, e.g., Karam et al., 2000; Sentürk et al., 2011; reviewed in Dastjerdi et al., 2012). It is not clear if any mutations disrupt cluster formation. For example, the homeobox transcription factor *Engrailed* (*En2*) is expressed by a cluster subset from about E15 but *En2* deletion does not significantly disrupt the adult zone-and-stripe pattern: rather it seems to have subtle (probably secondary) effects on lobulation and the formation of mossy fiber afferent topography—Millen et al., 1994; Kuemerle et al., 1997; Sillitoe et al., 2010). One candidate for primary patterning defects is the lysosomal acid phosphatase null mutant (*Acp2-/-;* a.k.a. *Nax*), which appears to have missing stripes in both the AZ and CZ, consistent with abnormal cluster formation, but other explanations are equally plausible (Bailey et al., 2014). The boundaries between adjacent transverse zones are also apparent during this same temporal window (e.g., Ozol et al., 1999; Fujita et al., 2012; Armstrong and Hawkes, 2014). Thus, it is clear that the fundamental patterning of the cerebellar cortex—the bud of the cerebellar rose—is already established at birth.

### From Clusters to Stripes

The transformation of the embryonic PC cluster array into the adult zone-and-stripe architecture occurs between E18 and P20. The transformation of the clusters (PCs plus affiliated interneurons, afferent terminals) into stripes is triggered by Reelin (Reln) signaling. Reln is a glycoprotein secreted in the cerebellar cortex by neurons of the rhombic lip migratory stream (reviewed in Nimura et al., 2019). Reln binds to a receptor complex on the PC surface comprising ApolipoproteinE Receptor 2 (ApoER2) and the Very-Low Density Lipoprotein Receptor (VLDLR: Trommsdorff et al., 1999; Hiesberger et al., 1999). The receptor signal is transduced via the phosphorylation by two tyrosine kinases—*Src* and *Fyn* (Howell et al., 1999) of the intracellular adaptor protein Disabled1 (*Dab1*: Howell et al., 1997: and its alleles *scrambler* (*Dab1^scm^*) and *yotari* (*Dab1^yot^*), Sheldon et al., 1997). The upshot is probably that PC-PC homophilic binding in the clusters is downregulated (as has been suggested for the cortex, Hoffarth et al., 1995), thereby facilitating cluster dispersal. The clusters disperse predominantly along the anteroposterior axis—roughly a 25-fold lengthening in the vermis—and thus the clusters in each transverse zone become the adult stripe arrays.

In mutations that block the Reln→Dab1 signaling pathway the PC clusters fail to form stripes and remain as ectopic clumps at the core of the cerebellar anlage. This was first reported in the naturally occurring ataxic mouse mutant *reeler* (*Reln*^rl^; Falconer, 1951). Subsequently, the same reeler phenotype was found due to other mutations of elements that comprise the Reln→Dab1 pathway: for example, the double-deletion of *Apoer2/Vldlr* (Trommsdorff et al., 1999), the deletion of *Dab1* (Howell et al., 1997) and double mutants of *Fyn* and *Src* (Howell et al., 1999; reviewed in D’Arcangelo, 2014). In addition of these mutations, which disrupt all PC cluster dispersal, other mutations act selectively on PC subsets to yield patterned disruptions of the cerebellar cortex. The usual description of the Reln→Dab1 pathway assumes that Apoer2 and Vldlr combine in individual PCs to form a functional Reln receptor complex (Trommsdorff et al., 1999). Consistent with this, the *Apoer2−/−:Vldlr −*/− double null has a full *reeler* phenotype. However, single null mutations show more complex phenotypes (Larouche et al., 2008). The single *Apoer2−/−* (a targeted disruption of *Apoer2* by a pol2*neo* cassette) has a few specific ectopic PC clusters, largely restricted to zebrin II- PCs located near the midline. Likewise, *Vldlr-/-* mice (with a disruption of exon 5) have only a small subset of ectopic PC clusters, some zebrin II +, others zebrin II-, suggesting differential subreceptor expression by PC subsets and hence patterned ectopias in the null phenotypes. However, while no PC ectopia is present in mice heterozygous for either receptor alone mutants double heterozygous for the two receptors (*Apoer2* ± :*Vldlr* ±) have a single small cluster of ectopic zebrin II- PCs.

PC cluster dispersal phenotypes are common. In addition to the full *reeler* phenotype in which all PC clusters fail to disperse, several naturally occurring mouse mutants show PC cluster dispersal phenotypes that are restricted to specific transverse zones. The best-understood are *rostral cerebellar malformation*, a spontaneous mutation of the netrin receptor gene (*Unc5c^rcm^*, Ackerman et al., 1997), and *cerebellar deficient folia* (a mutation at the catenin alpha 2 (*Ctnna2)* locus, Beierbach et al., 2001). Both phenotypes show a massive failure of PC cluster dispersal in the AZ while the posterior clusters are only mildly affected (Eisenman and Brothers, 1998; Goldowitz et al., 2000). Selective failures of cluster dispersal are also seen in the *weaver* mouse (*Kcnj6^wv^*: a mutation in the inwardly rectifying potassium channel GIRK2, Surmeier et al., 1996). In particular, the *wv/wv* homozygote shows a very specific PC dispersion defect in which a subset of zebrin II + /HSP25 + embryonic clusters located in the CZ fails to disperse normally and remains ectopic in their embryonic configuration (Armstrong and Hawkes, 2001).

Finally, multiple PC death mutants have been identified with patterned phenotypes that are restricted by the zone-and-stripe architecture. For example, a transgenic model of Niemann-Pick disease type A/B (an acid sphingomyelinase knockout (*Smpd1^tm1Esc^*), Sarna et al., 2001) shows the selective loss of zebrin II- PCs. In most cases, PC death is postnatal, and the surviving PCs remain in their normal stripe and zone locations, indicating that the defect has no effect on pattern formation (i.e., a “patterned” defect rather than a “patterning defect”: reviewed in Sarna and Hawkes, 2003). However, mutations that lead to embryonic PC death are less straightforward to interpret. For example, null mutations of *Ebf2* show a 30–40% loss in PCs, which happens prenatally (Croci et al., 2006, 2011). The result is a cerebellar hypoplasia with reduced lobulation, but more-or-less normal zone-and-stripe patterning, implying that the shortfall in PC numbers occurs during neurogenesis but before patterning begins [e.g., in the early VZ to cerebellar plate stage (E10-E11); also, see Kuemerle et al., 1997].

Whether PC death plays a role in refining the embryonic zone and patch pattern is unclear. On the one hand, here is evidence that PC death is concentrated at perinatal zone-and stripe boundaries, notably at the midline, consistent with a role in sculpting stripes and eliminating positional errors (Jankowski et al., 2011). In addition, there is some support for the idea that embryonic PC death might be transverse zone- or cluster-specific. For example, in the perinatal *Ebf2* null mouse, substantial embryonic PC apoptosis is concentrated in the AZ (Croci et al., 2011). However, suppression of perinatal PC death by deletion of the apoptosis-regulator BCL-Associated X-Protein (*Bax-/-)* results in a hypertrophic cerebellar cortex with 30% additional PCs but no patterning abnormalities (reviewed in Vogel, 2002), consistent with a role for apoptosis in size regulation rather than editing and fine tuning.

## Patterning Mutations in the Excitatory Granule Cell Development Pathway

The patterning of the granule cell pathway happens in parallel to the PC VZ→cerebellar plate→cluster array→stripe formation pathway (a thorough review of granular layer patterning, together with useful cartoons, can be found in Consalez et al., 2021). All granule cells of the cerebellar cortex derive from *Atonal Transcription Factor 1* (*Atoh1:* a.k.a *Math1*) lineage in the URL, and deletion of *Atoh1* completely ablates the granular layer (Akazawa et al., 1995; Ben-Arie et al., 1997; reviewed in Consalez et al., 2021). GCPs migrate from the URL, starting at E10-E12, and proliferate and spread to cover the entire surface of the cerebellar anlage as the external granular layer (EGL) by E18. Starting around E18 postmitotic granule cells descend ventrally from the EGL, guided by Bergmann glial fibers through the dispersing PC clusters and the developing molecular layer, and settle in the nascent granular layer. Populating the granular layer proceeds until P20-P30, by which time the GCPs are spent, and the EGL has disappeared.

Within the URL, before the EGL is formed, there is already elaborate patterning as revealed by the expression of multiple transcription factors (Yeung et al., 2014), and by E18, the patterning of the EGL is established. Four EGL transverse domains that derive from distinct GCP populations are revealed by differences in gene expression, birthdates, lineage restriction and the consequences of mutations (reviewed in Consalez et al., 2021). Each transverse domain is aligned with its counterpart in the PC layer—the EGL-AZ (eAZ), eCZ, ePZ, and eNZ. This alignment presumably arises because the PC cluster architecture restricts the dispersal of different GCP lineages or the underlying PC clusters induce gene expression in the overlying EGL (e.g., Smeyne et al., 1995). In particular, multiple mutations result in defects with phenotypic abnormalities that are restricted at EGL transverse domain boundaries. These phenotypes are of two kinds: intrinsic defects that directly affect the GCPs [e.g., *meander tail* (*mea/mea*), Hamre and Goldowitz, 1997] and extrinsic defects due to abnormalities in the local environment (e.g., defective GCP production in sonic hedgehog (*Shh*) null mice, Lewis et al., 2004; PC defects in the Retinoid receptor-related Orphan Receptor (ROR)α mutant *staggerer* (*Rora^sg^*), reviewed in Gold et al., 2003; Vitalis and Mariani, 2018). These are often difficult to distinguish in practice, and both intrinsic and extrinsic effects are often present concomitantly (e.g., *weaver*, Smeyne and Goldowitz, 1989).

The eAZ/eCZ boundary restricts the expression of several genes. For example, expression the homeodomain transcription factor *Otx1* (Frantz et al., 1994), fibroblast growth factor alpha, Zic1 (Aruga et al., 1998), and protein tyrosine phosphatase rho (McAndrew et al., 1998), are all restricted predominantly to the EGL anterior to the eAZ/eCZ boundary. In contrast, expression of *Otx2* (Frantz et al., 1994) and the homeodomain transcription factor *Lmx1a* is restricted to the EGL posterior to the eAZ/eCZ boundary (and see below for the eCZ). The eAZ/eCZ is also a lineage-restriction boundary in embryonic stem cell chimeras, and hence in this case at least the EGL boundary is not secondary to induction by the underlying PC clusters (Hawkes et al., 2008).

Several mutant EGL phenotypes are restricted at the eAZ/eCZ boundary. The most prominent phenotype is the presence of an agranular AZ. This is seen in the *meander tail (mea/mea)* mutant (Ross et al., 1990), in *rostral cerebellar malformation* (*Unc5c^rcm^*, Ackerman et al., 1997; Eisenman and Brothers, 1998), in the failure of the zebrin II- PC subpopulation in the cadherin receptor mutant *cerebellar deficient folia* (*Ctnna2^cdf^*, Beierbach et al., 2001) and in the heterozygous *weaver* mutant (*wv/*+ , Eisenman et al., 1998; Armstrong and Hawkes, 2001). In contrast, a null mutation of the transcriptional activator gene *Neurogenic Differentiation 1* (*Neurod1*) results in a selective loss of GCPs posterior to the eAZ/eCZ boundary (Miyata et al., 1999; Cho and Tsai, 2006). As noted above, in the posterior EGL the GCPs are Lmx1a+, and consequently *Lmx1a* mutations (for instance, the deletion in the autosomal recessive mutant *dreher* (*Lmx1a ^drJ^*)) manifest EGL defects focused on the posterior vermis (Chizhikov et al., 2010: interestingly, in null mutations of *Lmx1a*, GCPs over-migrate into the anterior vermis, leading to posterior vermis hypoplasia). In addition, in the *dreher* mouse both roof plate and rhombic lip defects induce extreme deformities in the overall morphology including dramatic vermis distortion (Millonig et al., 2000; Chizhikov et al., 2010), presumably secondary to the switching of the *Lmx1a* + GCPs to an anterior (*Lmx1a-*) fate. However, despite the gross cortical deformities the underlying zone-and-stripe architecture is preserved (e.g., the HSP25 ± and zebrin II ± stripe arrays are intact, Sillitoe et al., 2014). Similarly, *Ebf2* is expressed transiently in the URL and the *Atoh1* + migratory stream between E12.5-E13.5 (Croci et al., 2006, 2011; Chung et al., 2008). Genetic fate mapping indicates that granule cells derived from *Ebf2* + precursors are restricted largely to the eAZ [Badaloni et al., 2019: the AZ is populated predominantly by early born GCs (Machold and Fishell, 2005), so this may account for their anterior location]. There is no obvious phenotype in the *Ebf2−/−* EGL. Finally, in *Dab1* null mutants a clear separation of anterior and posterior EGLs is found at the eAZ/eCZ boundary presumably secondary to the failure of PC cluster dispersal (Gallagher et al., 1998).

The evidence for a distinct eCZ/ePZ boundary and a specific eCZ domain is less clear cut. First, in chimeras of wildtype and the mutation *small eye* (*Pax6*^Sey/Sey^) a lineage boundary is seen in lobules VII where the *Pax6* mutation preferentially affects the EGL anterior to the eCZ/ePZ boundary (Swanson and Goldowitz, 2011). Secondly, *Otx1/Otx2* expression reveals two boundaries in the EGL—the eAZ expresses only *Otx1* and the ePZ expresses *Otx2*. However, between the two the eCZ is revealed as a transverse domain of Co-expression (Frantz et al., 1994).

No *positive* markers of the third EGL transverse domain, the ePZ, are known. However, the ePZ can be defined as located between the posterior border of the eCZ and the anterior border of the eNZ. This is also a lineage boundary in chimeras at the same approximate location (Hawkes et al., 2008) and an expression boundary for zebrin II in the heterozygous mutant *Lurcher* (Tano et al., 1992), which has constitutive activation of the delta 2 ionotropic glutamate receptor (*Grid2^Lc^*, Wollmuth et al., 2000). The posterior boundary of the ePZ is revealed by several positive markers of the eNZ. The clearest example is the restricted expression of the homeodomain transcription factor gene *Tlx3*, which clearly delineates the fourth EGL sub-domain aligned with the PC NZ (Logan et al., 2002). Expression of a neurotrophin-3-lacZ transgene is similarly restricted to the adult granular layer of the NZ, both during development and in the adult (Tojo et al., 1995), and the same boundary is seen when an *Atonal-cre* was used to eliminate *Neurod1* (Pan et al., 2009). Finally, a transverse discontinuity in the EGL is seen at the same approximate location in the heterozygous *weaver* mutant (*wv/*+, Eisenman et al., 1998; Armstrong and Hawkes, 2001).

The presence of transverse domains in the EGL is clear but evidence for parasagittal stripes is much less so. One candidate is in the young rabbit, where a monoclonal human granulocyte antibody B4,3 (= anti-CD15) reveals a striped expression pattern in the EGL (Marani and Tetteroo, 1983). Presumably this is a secondary induction via the underlying PC clusters or stripes. Another novel mediolateral pattern is seen in the evidence that one subgroup of GCPs in humans—concentrated in the hemispheres—is especially sensitive to *Shh* signaling and, perhaps as a result, is also more likely to give rise to meduloblastomas (Tan et al., 2018): how, if at all, this GCP mediolateral segregation relates to the anteroposterior EGL compartmentation, is unknown.

## Summary and Conclusion

Mutant mice with cerebellar phenotypes have proved powerful tools to explore the mechanisms of pattern formation (Sidman et al., 1965). Abnormal patterns include missing cells, either never born (e.g., the *Zfp423*Δ*9-20* allele, Casoni et al., 2017; *Lmx1a−/−*, Chizhikov et al., 2010) or died (e.g., *Ebf2−/−*, Croci et al., 2006; Chung et al., 2008; selective granule cell death in *Neurod1−/−*: Miyata et al., 1999; Cho and Tsai, 2006), ectopias due to abnormal cell dispersal (e.g., for PCs, mutations in the Reln→Dab1 pathway or in *wv/wv*—Armstrong and Hawkes, 2001; for granule cells, examples include abnormal EGL migration in *Lmx1a−/−*, Chizhikov et al., 2010), and probably expression defects (“pattern defects” vs. “expression defects”; e.g., expression patterns induced by the local environment). These include 5′-nucleotidase expression by Bergmann glial cells induced by the local zebrin II + PCs (Scott, 1963; Eisenman and Hawkes, 1989) or abnormal tyrosine hydroxylase expression in *rolling mouse Nagoya* (*rol/rol*) a mutation in the α1 subunit of the calcium channel gene *Cacna1a* (Sawada et al., 1999), and the spontaneous *dilute-lethal* (*Myo5a^d–l^*) mutant (Sawada et al., 1999).

It is remarkable how much light has been shed on cerebellar patterning and the mechanisms of pattern formation through the analysis of mutant mouse strains. The mutant phenotypes reveal stages of patterning during development that become progressively complex. During PC generation and the formation of the cerebellar plate (E10–E13) there is at least some PC subtype specification, but whether the cerebellar plate contains a protomap is not known. From E14-E18, the cerebellar plate disperses into the embryonic PC cluster array. In parallel, during granule cell development, mutant phenotypes reveal reproducible boundaries within the EGL that arise often from distinct lineages in the URL (e.g., *rcm, cdf, mea* etc.). The transverse domains in the EGL and the zones in the PC layer become aligned during embryogenesis, but the mechanism that guides this is not well understood. As a result, by E18 the fundamental zone-and-stripe pattern is established. The embryonic bud is transformed into the adult flower during the first 3–4 postnatal weeks due to PC dispersal triggered by Reln→Dab1 signaling and granule cell migration from the EGL to the maturing granular layer. Finally, granular layer maturation reveals a further stage in cerebellar patterning—the subdivision of stripes into thousands of patches (e.g., Hawkes and Turner, 1994; Hawkes, 1997; Ozol and Hawkes, 1997; Sillitoe et al., 2003)—probably associated with inductive influences during the maturation of the mossy fiber projection maps (e.g., Shambes et al., 1978; Schilling et al., 1994).

## Author Contributions

The author confirms being the sole contributor of this work and has approved it for publication.

## Conflict of Interest

The author declares that the research was conducted in the absence of any commercial or financial relationships that could be construed as a potential conflict of interest.

## Publisher’s Note

All claims expressed in this article are solely those of the authors and do not necessarily represent those of their affiliated organizations, or those of the publisher, the editors and the reviewers. Any product that may be evaluated in this article, or claim that may be made by its manufacturer, is not guaranteed or endorsed by the publisher.
